# Presurgical structural connectivity predicts postsurgical cognitive impairment in glioma patients

**DOI:** 10.1093/braincomms/fcaf346

**Published:** 2025-10-24

**Authors:** Lars Smolders, Wouter De Baene, Karin Gehring, Remco van der Hofstad, Luc Florack, Geert-Jan Rutten

**Affiliations:** Department of Neurosurgery, Elisabeth-TweeSteden Hospital, 5022 GC Tilburg, The Netherlands; Department of Mathematics and Computer Science, Eindhoven University of Technology, 5612 AE Eindhoven, Netherlands; Department of Cognitive Neuropsychology, Tilburg University, 5000 LE Tilburg, Netherlands; Department of Neurosurgery, Elisabeth-TweeSteden Hospital, 5022 GC Tilburg, The Netherlands; Department of Cognitive Neuropsychology, Tilburg University, 5000 LE Tilburg, Netherlands; Department of Mathematics and Computer Science, Eindhoven University of Technology, 5612 AE Eindhoven, Netherlands; Department of Mathematics and Computer Science, Eindhoven University of Technology, 5612 AE Eindhoven, Netherlands; Department of Neurosurgery, Elisabeth-TweeSteden Hospital, 5022 GC Tilburg, The Netherlands

**Keywords:** machine learning, tractography, network hubs, network neuroscience

## Abstract

Glioma patients frequently suffer from cognitive impairments after surgery, but predicting these impairments preoperatively at an individual level remains challenging. Cognitive functions are increasingly studied from a network perspective, where an important role is played by the Default Mode Network (DMN) and Frontoparietal Network (FPN). Hypothesizing that postsurgical cognitive impairments arise from structural network vulnerabilities, we trained models using presurgical structural connectivity of DMN and FPN regions to predict postsurgical cognitive impairment. We obtained individualized structural connectomes in 63 glioma patients (grades II–IV) who underwent diffusion-weighted MRI before surgery (T0) and neuropsychological screening 3 months after surgery (T3) and, for a small majority, adjuvant treatment. Random forest classifiers were trained on a combination of baseline (sociodemographic and clinical), tumour location and structural network variables available before surgery to predict postsurgical cognitive impairment in individual patients. Classifier performance was measured as area under curve of the receiver operating characteristic (AUC-ROC), testing statistical significance via permutation testing. Predictor importance was calculated post-hoc using Shapley additive explanations for trees. Postsurgical impairment was predicted by baseline variables available at T0 (AUC = 0.69, *P* = 0.011), presurgical DMN degrees (AUC = 0.73, *P* = 0.001), presurgical FPN degrees (AUC = 0.73, *P* = 0.001) and combinations of network and baseline variables (AUC = 0.75, *P* < 0.001; AUC = 0.76, *P* < 0.001 for DMN and FPN, respectively), but not by tumour location only (AUC = 0.62, *P* = 0.068). The combination of baseline variables, DMN degrees and FPN degrees (AUC = 0.76, *P* < 0.001) did not improve results. Importantly, models including network variables performed better than models using baseline or tumour location variables only. The most important predictors of postsurgical cognitive impairment were older age and low connectivity of the left lateral superior frontal gyrus (DMN), right pars opercularis (FPN) and bilateral middle frontal gyrus (DMN). This study represents a step towards preoperative prediction of postsurgical cognitive impairments in individual glioma patients. Our results underscore the importance of the DMN and FPN for cognition and suggest a biomarker for cognitive resilience to damage from treatment. The success of our model illustrates the utility of individual structural connectomes for studying cognitive impairment. Future expansions, e.g. incorporating resting-state fMRI, could improve our model. Ultimately, a sufficiently accurate model could be applied in neurosurgical planning by assessing a patient’s risk of postsurgical impairment from presurgical information only, improving counselling of glioma patients regarding surgical expectations.

## Teaser

Glioma patients frequently suffer from cognitive impairments after surgery. Smolders *et al*. developed a model that predicts these impairments using properties of white matter tracts obtainable ‘before surgery’. The success of this model demonstrates the utility of individualized structural connectomes and suggests biomarkers for cognitive resilience to damage from treatment.

## Graphical Abstract

**Graphical Abstract:**
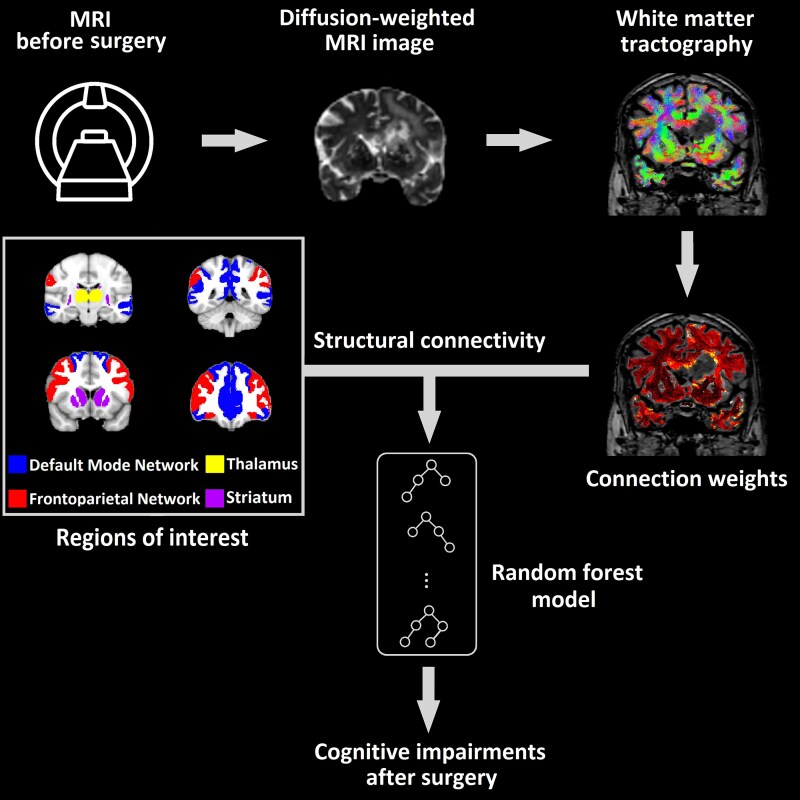

## Introduction

Glioma patients frequently experience neurological and cognitive impairments after surgery and subsequent adjuvant treatment.^1-3^ In some cases, the risk of developing impairments such as hemiparesis or visual field deficits can be estimated reasonably well based on anticipated surgical damage to specific cortical regions or white matter (WM) connections.^4^ However, such predictions are not accurate at individual patient level for ‘cognitive’ functions, such as working memory or complex reasoning.^5,6^ Furthermore, adjuvant therapies, such as chemo- and radiotherapy, also cause cognitive problems that are difficult to predict for individual patients.^7,8^ Between 25% and 50% of glioma patients experience a decline in at least one domain of cognitive functioning 3 months after initial treatment,^9,10^ highlighting the clinical relevance of understanding the relationship between brain structure and cognitive functioning for individual patients. The necessary next step in this line of research is a model that can predict postsurgical cognitive impairment in individual patients from presurgical information. Given such a model, clinicians can better counsel individual glioma patients about the risks and benefits of surgery and adjuvant treatment.

In the last two decades, the importance of a connectionist view on the brain in the context of neurosurgical decision-making has become clear.^11-13^ In this view, not only the function of individual brain regions is considered, but also the connections between them and the resulting interactions in the whole-brain network. The mathematical formalization of these concepts has given rise to the field of ‘network neuroscience’,^14,15^ in which the brain is modelled as a complex network of interconnected and interacting regions. Within the range of models studied in network neuroscience, a distinction is made between Structural Connectivity (SC), networks of regions anatomically linked by WM bundles, and Functional Connectivity (FC), where regions are related by functional co-activation. The properties of both these network types have been successfully leveraged for several applications, such as developing biomarkers for dementia,^16-18^ ADHD^19,20^ and schizophrenia,^21-23^ and studying the mechanisms of epilepsy.^24,25^

To date, the majority of studies investigating cognition in glioma patients have focussed on FC. An important finding from the study of FC in large groups of healthy subjects is the consistent emergence of ‘resting-state networks’, which are subdivisions of the whole-brain network that display more correlated activity within a network than between networks when a subject is at rest.^26,27^ Two of these networks, the Default Mode Network (DMN) and the Frontoparietal Network (FPN) are regularly implicated in cognitive functioning and impairment.^28,29^ The DMN is found to be most active when a subject is at rest and deactivates in relation to other networks when focussing on a task.^30^ Abnormal activity in and impaired deactivation of the DMN is observed in glioma patients^31-33^ and altered FC in the DMN was found to correlate with post-treatment cognitive functioning.^34^ The FPN is associated with cognitive control and is thought to be responsible for DMN deactivation during task performance.^29,35^ Unsuccessful reorganization of the FPN during a working memory task is associated with poor cognitive performance in glioma patients.^36^ Furthermore, altered interaction between the DMN and FPN is related to reduced working memory in this patient group.^37^

The functioning of these resting-state networks is strongly dependent on the physical connections between their regions, i.e. on the SC of these networks. Structural connections support information transfer and long-range interactions, which are required for these distributed networks to function properly.^38,39^ A small number of studies have investigated SC in glioma patients before surgery. For example, it has been shown that gliomas impact SC in both local^40-42^ and remote^43^ areas of the brain. Furthermore, disruptions in structural integrity, a measure of SC that estimates the alignment of fibres in a WM bundle, are correlated with low cognitive performance,^41,43^ and higher structural integrity in the contralesional hemisphere has been associated with better survival, potentially suggesting a compensatory mechanism.^43^ The topology of structural networks in glioma patients also differs from that of healthy subjects, exhibiting a higher degree of small-world organization and altered hub structure.^44,45^ Features of the structural connectome of glioma patients can predict performance status^45^ and aphasia.^46^ Furthermore, postoperative cognitive deficits can be predicted from postoperative SC,^47^ based predominantly on structural connections between cortical regions of different resting-state networks (such as the DMN and FPN) rather than connections between regions within a single network, and on connectivity involving hub nodes.^47^ While not often explicitly studied in most research on glioma patients, the ‘thalamus’ is an important subcortical structure that structurally connects the entire cortex (including the DMN and FPN),^48-50^ and has been shown to support a wide range of cognitive functions in healthy subjects.^51,52^ Furthermore, the importance of the striatal subcortical regions in frontoparietal connectivity has recently been highlighted, indicating a role in integrating the DMN and FPN.^53-55^

Drawing from these observations, we hypothesized that the structural integrity of connections with Regions of Interest (ROIs) in the DMN, in the FPN and subcortical regions plays an important role in the postsurgical cognitive ability of glioma patients. While it has been convincingly shown that network connectivity is related to cognition at a group level, the organization of the human connectome varies strongly between individuals, even in healthy subjects, in terms of both FC^56,57^ and SC.^58^ This inter-individual variance is even higher in glioma patients, where the effects of plasticity play an important role, as highlighted by earlier connectomic studies,^59-61^ presurgical language fMRI,^62,63^ and direct electric stimulation during awake surgery.^11,64^ Therefore, group-level connectivity templates are inherently unreliable for studying individual glioma patients, and an individual-level mapping of the connectome is required to accurately study the glioma patient’s brain.

To best aid in presurgical decision-making, a predictive model should accurately identify patients at risk of postsurgical cognitive impairment using only presurgical information. If such a model proves to be accurate, clinicians can make better informed decisions about treatment options tailored to each individual patient. The goal of this study was to develop and validate a model that predicts postsurgical cognitive impairment from presurgical baseline variables and presurgical SC of the DMN, FPN and subcortical regions extracted from individual connectomes of 63 glioma patients.

## Methods

### Subjects

We retrospectively considered all glioma patients that underwent resective tumour surgery and diffusion-weighted MRI between July 2016 and July 2023 in the Elisabeth-Tweesteden Hospital (ETZ, Tilburg, The Netherlands) for inclusion in this study. Inclusion criteria were (i) histologically confirmed glioma WHO grades I–IV, (ii) availability of T1-weighted and diffusion-weighted MRI data in the week before surgery (T0) and 3 months after surgery (T3) and (iii) availability of neuropsychological screening data at T0 and at T3. Exclusion criteria were (i) age below 18, (ii) history of intracranial surgery, (iii) history of cranial radiotherapy or chemotherapy and (iv) history of psychiatric or neurological disorders. This study was part of a protocol registered with the Medical Ethics Committee Brabant (file number NW2020-32). Ethical approval for this project was obtained from the University Ethics Review Board (ERB) of the School of Social and Behavioural Science of Tilburg University (RP398). All procedures were carried out with written informed consent of all subjects and in accordance with the principles of the Declaration of Helsinki.

### Image acquisition

MRI images were acquired using a Philips Achieva 3T MRI scanner (Philips Medical Systems, Best, The Netherlands) using a standard 32-channel radiofrequency head coil. High-resolution whole-brain anatomical images were acquired using a T1-weighted sequence (TR/TE: 8.4/3.8 ms, FOV: 254 × 254 × 158 mm, flip angle: 8°, sagittal slice orientation, voxel size 1 mm isotropic) and FLAIR sequence (TR/TE: 11 s/125 ms). DWI images were acquired using an echo-planar imaging (EPI) sequence (TR = 8 s; TE = 115 ms; 6 b = 0 volumes, 50 b = 1500 volumes, 2 mm isotropic voxel size).

### Image preprocessing

T1 images were skull-stripped using HDbet^65^ to enable affine registration to DWI space. DWI masks for subsequent processing were automatically generated using *dwi2mask* as implemented in MRtrix3.^66^ We observed that many masks were missing parts of the orbitofrontal cortex. Since incorrect masks could affect processing steps further down the processing pipeline, we registered the mask resulting from skull-stripping the T1 images onto the DWI images as a substitute for initial DWI masking using affine registration performed with ANTs (https://github.com/ANTsX/ANTs). These masks resulted in correct coverage of the entire WM and grey matter (GM), improving the quality of the subsequent processing. We denoized DWI images using *dwidenoise*, corrected for EPI distortion and Eddy currents using *dwifslpreproc*, and corrected for bias fields using *dwibiascorrect*, all implemented in MRtrix3. Since no b0 with reverse phase encoding was acquired, we applied nonlinear registration of an inverted contrast skull-stripped T1 image^67^ to each b0 image using ANTs to most accurately register T1 images to DWI images for later parcellation transfer. Finally, all DWI images were resampled to 1 mm isotropic voxel size. All steps of the preprocessing pipeline were manually checked.

The contrast-enhancing parts of glioma were automatically segmented using an nnU-Net convolutional neural network^68^ that was trained on T1, T1c and FLAIR scans from the BraTS dataset.^69,70^ Segmentations were visually inspected and manually corrected wherever necessary. Tumour volume was calculated by summing segmentation voxel volumes. Tumour location was categorized using binary variables encoding overlap with hemispheres and lobes (frontal, parietal, temporal, occipital and insular).

### Tractography

We used single-shell 3-tissue spherical deconvolution (SS3T-CSD)^71,72^ as implemented in the MRtrix3Tissue fork (https://3Tissue.github.io) of MRtrix3 to estimate fibre orientation distributions (FODs) in WM. Briefly, CSD allows the estimation of multiple fibre orientations within a single voxel,^73^ as opposed to the widely clinically used diffusion tensor imaging model.^74,75^ SS3T-CSD is an extension of CSD that estimates FODs for WM, GM and cerebrospinal fluid (CSF) separately using only a single shell of DWI data. This way, the signal from WM fibres, which is the signal of interest in tractography, can be isolated from mixed diffusion signal in voxels containing multiple tissue types. Before FOD estimation, WM response functions generated by SS3T were averaged over all patient scanning sessions to ensure that estimated FOD amplitudes were comparable between patients and between sessions.^76^

To guarantee anatomically plausible terminations of streamlines (i.e. starting and ending in the boundary between WM and GM and passing through only WM) we applied Anatomically Constrained Tractography,^77^ which depends on a 5-tissue type segmentation of the anatomical T1 image co-registered to the diffusion image. Briefly, the T1 image is automatically segmented into WM, GM, CSF and deep GM (e.g. thalamus, basal ganglia). The fifth tissue type corresponds to pathological tissue, where no assumptions regarding seeding, terminating or tracking are upheld. However, this also means that no streamlines are seeded inside glioma tissue, which, especially in the case of LGG, can cause a severe underestimation of tracts in and around the tumour. Since conventional 5-tissue type segmentation fails inside tumour tissue due to the grey-appearing nature of glioma tissue, we derived a tissue segmentation from DWI data, using the WM, GM and CSF estimations from SS3T-CSD.^78^ This way, streamlines are seeded inside tumours whenever the diffusion data indicates the presence of a WM–GM boundary.

Tractography was performed using the iFoD2 algorithm as implemented in MRtrix3, seeding 4 000 000 streamlines in the GM–WM boundary of the brain, enabling the backtracking option to re-track streamlines that do not terminate in WM–GM boundary voxels. We set the cutoff value for FOD amplitude to 0.06 to allow streamlines to pass through WM infiltrated by tumour tissue. Finally, the minimum streamline length was set to 10 mm and the maximum streamline length was set to 250 mm.

### Image parcellation

To obtain personalized networks for each patient, an accurate parcellation of the brain into ROIs was needed. Frequently used methods, such as surface mapping with Freesurfer, usually fail when applied to subjects with large lesions,^79^ including large brain tumors.^80^ Therefore, we opted to parcellate patient scans using SLANT,^81^ a segmentation tool based on a convolutional neural network. This deep learning model has not been trained on brain tumour patients but has shown a surprisingly high accuracy on presurgical glioma patients in practice.^82^ The segmentations were then nonlinearly registered to DWI space by applying the transformations obtained according to the procedure described above in ‘Image preprocessing’.^67^ We extracted cortical GM, subcortical GM, and brain stem ROIs from the resulting segmentations. SLANT subdivides the striatum into the bilateral nucleus accumbens, caudate nucleus and putamen, but does not subdivide the thalamus, resulting in eight subcortical GM regions. In total, each patient scan was parcellated with 115 ROIs.

### Structural connectivity

To measure the strength of connections between ROIs, we used the spherically informed filtering of tractograms (SIFT2) algorithm^83,84^ to weight streamlines in the whole-brain tractogram in proportion to their contribution to the intra-axonal diameter, based on individual patient DWI data. This metric is assumed to roughly capture the capacity for information transfer in a tracked WM bundle when one sums the weights of the constituting streamlines. While many studies use either the number of streamlines or metrics of structural integrity such as FA or MD to quantify SC, each of these have severe limitations and questionable interpretations in and around lesions (see Jones *et al*.^85^ for an overview). In contrast, SIFT2 has been shown to accurately calculate connectivity in the presence of lesions^84,86,87^ and has high reproducibility across scanning sessions.^88-90^ For each pair of ROIs, we summed the SIFT2 weights of all streamlines starting or ending in either ROI, using a 4 mm radial search around streamline terminations.^89^ To preserve all information present in the weighted edges, we chose to not threshold the resulting SC matrices into binary graphs. Unthresholded connectomes are argued to contain more biologically relevant information than binary connectomes,^91,92^ based on for instance the heterogeneity in connection density observed in tract-tracing studies^93^ and the importance of weak connections.^94^

### Network features

To reduce the dimensionality of the data (i.e. 115 × 114/2 = 6555 unique edges per patient) we sought to extract meaningful features from the SC matrices. To do this, we focused on summary statistics calculated on nodes that are part of the DMN, FPN, thalamus and striatum.

**Figure 1:**
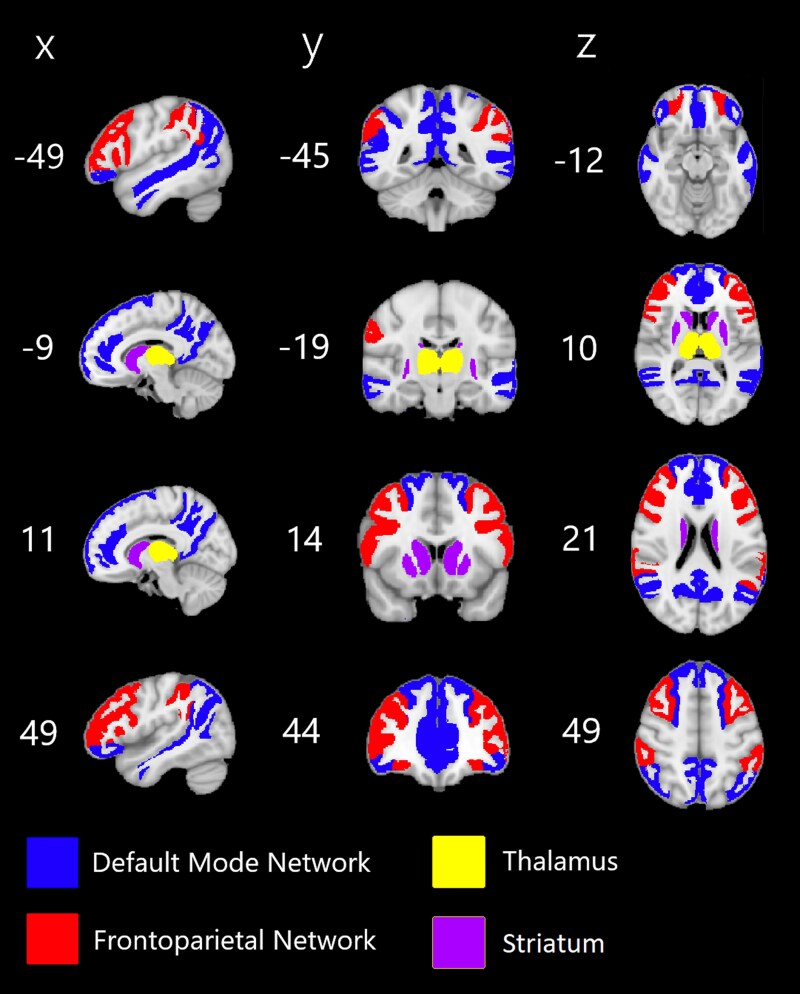
**Overview of ROIs considered in this study.** ROIs are extracted from a SLANT brain segmentation of the Montreal Neurological Institute (MNI) T1-weighted image. Columns show sagittal, coronal and axial slices with corresponding MNI coordinates, respectively.

Firstly, we identified ROIs in the SLANT parcellation that corresponded to the DMN and FPN by calculating each SLANT parcel’s overlap with definitions of the DMN and FPN found in Yeo’s 7-network atlas.^26^ For each ROI in the SLANT parcellation, we assigned it to the network with which it had maximal overlap, if any. Furthermore, we added the thalamus and striatal regions to both the DMN and FPN subsets, since these subcortical areas integrate regions in the entire cortex. The resulting assignments are displayed in Fig. 1. The DMN ROIs consisted of the frontal pole, lateral orbital gyrus, medial frontal cortex, medial and lateral superior frontal gyrus, pars orbitalis, anterior and posterior cingulate gyrus, angular gyrus, precuneus, middle temporal gyrus, and left superior temporal gyrus. Bilaterally, these add up to 23 ROIs. The FPN ROIs consisted of the anterior orbital gyrus, middle frontal gyrus, pars opercularis, pars triangularis, and supramarginal gyrus, adding up to 10 ROIs.

The most straightforward ROI statistic is the ‘degree’ of each node, also called ‘degree centrality’, which is equal to the sum of all edge weights connected to a node.^95^ For each node in the DMN and FPN sets (including bilateral subcortical regions), we separately summed the weights of all edges connecting each node to the rest of the brain, resulting in 33 and 20 predictive variables per set, respectively. Note that we included connections to ROIs outside of the DMN and FPN in this calculation, i.e. we calculated the ‘whole-network’ degree of each ROI.

### Cognitive measures

A neuropsychological screening battery was administered in every patient as part of standard clinical care, using the computerized CNS Vital Signs.^96^ Tests were conducted on the same day at which patients underwent MRI scanning, both at T0 and at T3. The CNS VS screening battery consists of tests for verbal memory recognition, visual memory recognition, symbol digit coding, the Stroop effect, shifting attention, continuous performance, motor speed and reaction time. Detailed descriptions of cognitive tests and scoring are given in Supplementary Materials 1, Supplementary Table 1.

Each of the nine test scores were standardized to account for the effects of age, education and sex in a representative norm group of healthy individuals,^97^ resulting in a *Z*-score on each test for each patient. We labelled patients as ‘impaired’ if they scored *Z* < −1.5 in at least two test variables and ‘unimpaired’ otherwise.

### Baseline sociodemographic and clinical variables

Baseline variables (available at T0) used in predictive models were (i) sex, (ii) age, (iii) education level according to the Dutch Verhage scale,^98^ (iv) tumour volume, (v) binary variables representing tumour overlap with the frontal, parietal, temporal, occipital and insular lobes, and (vi) cognitive impairment (i.e. two or more cognitive *Z*-scores below −1.5) at T0 assessment. Variables added to predict impairment at T3 from T3 predictors were (i) administration of chemotherapy between T0 and T3, (ii) administration of radiotherapy between T0 and T3 and (iii) tumor grade classification.

Glioma diagnoses were graded into grades I–IV based on a combination of histopathological and molecular tumour characteristics according to the WHO grading scheme proposed in 2016.^99^ Diagnoses were classified as Low-Grade Glioma (LGG) or High-Grade Glioma (HGG). All WHO grade III and grade IV gliomas were classified as HGG. Isocitrate dehydrogenase (IDH)-mutated WHO grade II gliomas were classified as LGG, while IDH-wildtype grade II tumours were classified as HGG. IDH mutation status was determined based on immuno-histochemical testing in all patients, but further molecular determination was only performed on patients aged below 55 years with WHO grade II tumours and negative immune-histochemical determination in this retrospective data set.

### Prediction of cognitive impairment

We trained random forest classifiers as implemented in ‘scikit-learn’^100^ to predict cognitive impairment at T3 from predictors available at T0. Different sets of predictors were used, separated into T0 DMN ROI degrees, T0 FPN ROI degrees, baseline predictors available at T0, and combinations thereof.

The maximum tree depth was set to 3 to limit the risk of overfitting. Models were trained across 100 cross-validation loops, randomly splitting the patient group into 75% training data and 25% validation data in a class-stratified way. Predictor performance was assessed using the average area under the receiver operating characteristic curve (AUC-ROC) across cross-validation loops. Additionally, we calculated precision, recall, sensitivity and specificity of model predictions to investigate types of prediction errors in more detail. We assessed which predictors were most important in the fitted models by calculating Shapley additive explanations (SHAP) for tree models.^101^ These values quantify both the importance of features and the direction in which they have influence, i.e. we can use them to infer whether higher or lower values of a variable predict impairment.

To verify whether the predictive information of SC variables goes beyond mere tumour location, we also derived a granular set of location predictors using the SLANT atlas regions. For each ROI in the atlas, the percentage overlap with each patient’s tumour segmentation was calculated, measuring the proportion of each ROI’s volume that is covered by tumour tissue. Together, these location variables were used as a separate predictor set for cognitive impairment at T3.

To further validate the predictive power of SC features in glioma patients, we trained random forest models to predict cognitive impairment at T0 from SC at T0 and baseline variables available at T0 (excluding impairment at T0 itself), and predicted cognitive impairment at T3 from SC at T3 and clinical variables available at T3.

The code used to generate SC matrices and to predict cognitive impairment is shared on Github at https://github.com/larssmolders/predicting-postop-impairment.

### Statistical analysis

Statistical significance of predictor performance was assessed by permutation testing, randomly shuffling outcome variables 1000 times and repeating the cross-validation procedure using the same folds. We corrected *P*-values for multiple testing by applying a False Discovery Rate using the Benjamini–Hochberg procedure.^102^

## Results

### Patient characteristics

Sixty-three patients were retrospectively included in this study. Patient characteristics are described in Table 1.

**Table 1:** Patient characteristics

Patient characteristics	All patients (*n* = 63)
*Age (mean, std)	47.3 ± 15.4
*Female (*n*, %)	24 (38.1%)
*Education (mean, std)	5.5 ± 0.9
Tumour grade	
LGG (*n*, %)	34 (54.0%)
HGG (*n*, %)	29 (46.0%)
*Tumour hemisphere	
Left (*n*, %)	28 (44.4%)
Right (*n*, %)	36 (57.1%)
Bilateral (*n*, %)	1 (1.6%)
*Tumour lobe	
Frontal (*n*, %)	30 (47.6%)
Parietal (*n*, %)	16 (25.4%)
Temporal (*n*, %)	25 (39.7%)
Insular (*n*, %)	18 (28.6%)
Occipital (*n*, %)	2 (3.2%)
*Tumour volume (cm^3^, mean, std)	56.0 ± 54.1
Adjuvant treatment between T0 and T3	
Radiotherapy (*n*, %)	37 (58.7%)
Chemotherapy (*n*, %)	35 (55.6%)
*T0 impaired (*n*, %)	30 (47.6%)
T3 impaired (*n*, %)	34 (54.0%)
T0–T3 declined (*n*, %)	21 (33.3%)

*Used as baseline predictor variables.

**Figure 2:**
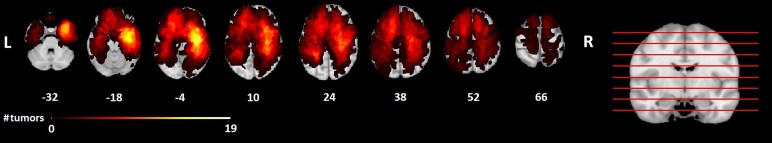
**Distribution of tumour locations across all patients normalized to MNI space.** MNI *z* coordinates of the axial sections are given.

The distribution of tumour locations across all patients is presented in Fig. 2. We observe the highest concentration of tumours in the right temporo-insular region.

### Predicting cognitive impairment

Predicting T3 impairment from T0 predictors yielded the results presented in Table 2. Baseline variables, DMN degrees, FPN degrees and all combinations of variables achieved significant performances, where the highest performance was achieved in combining baseline variables and network variables. The combination of DMN and FPN degrees did not improve results. Finally, tumour location only did not significantly predict cognitive impairment at T3. From the additional performance metrics computed (precision, recall, sensitivity, specificity), we see that including connectivity variables improves performance in all aspects, but especially specificity. This means models with connectivity variables are better at correctly identifying patients that have good postsurgical cognitive outcomes.

**Table 2:** Model performance predicting cognitive impairment at T3 from T0 connectivity and clinical variables.

Predictors	AUC (corrected *P*)	Precision	Recall	Sensitivity	Specificity
Baseline variables	**0.69 (*P*** **=** **0.011)**	**0**.**68**	**0**.**71**	**0**.**61**	**0**.**56**
Location variables	0.62 (*P* = 0.068)	0.59	0.69	0.58	0.45
T0 DMN degrees	**0.73 (*P*** **=** **0.001)**	**0**.**71**	**0**.**70**	**0**.**64**	**0**.**62**
T0 FPN degrees	**0.73 (*P*** **=** **0.001)**	**0**.**72**	**0**.**68**	**0**.**62**	**0**.**63**
Baseline var. + T0 DMN degrees	**0.75 (*P*** **<** **0.001)**	**0**.**73**	**0**.**74**	**0**.**67**	**0**.**63**
Baseline var. + T0 FPN degrees	**0.76 (*P*** **=** **0.001)**	**0**.**74**	**0**.**72**	**0**.**66**	**0**.**66**
Baseline var. + T0 DMN + FPN degrees	**0.76 (*P*** **<** **0.001)**	**0**.**73**	**0**.**72**	**0**.**66**	**0**.**64**

Values in bold represent statistically significant performance (*P* < 0.05, FDR corrected).

The results of predicting T0 impairment from T0 predictors and predicting T3 impairment from T3 predictors are presented in the Supplementary Materials 2 (Supplementary Tables 2 and 3, respectively). In both cases, impairment could be predicted from baseline predictors as well as from SC variables. Additionally, we tested whether combining the granular location variables with each of the models presented in Table 2 would affect performance, but found no improvements (Supplementary Table 4).

### Predictor importance

**Figure 3:**
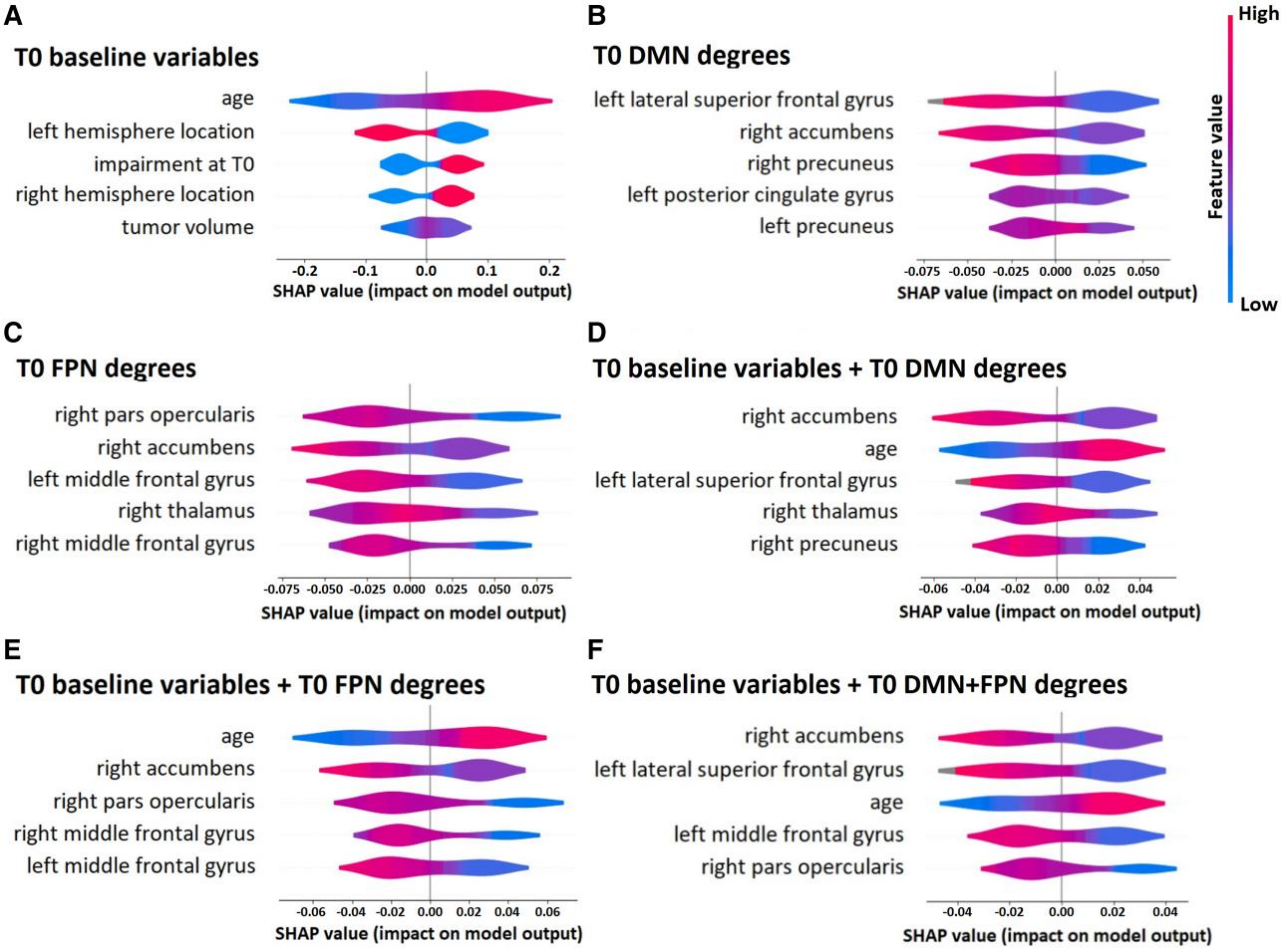
**SHAP scores for predictor sets.** The top five variables used in predicting T3 impairment from variables available at T0 are presented for models using (**A**) baseline variables, (**B**) DMN degrees, (**C**) FPN degrees, (**D**) baseline variables and DMN degrees, (**E**) baseline variables and FPN degrees, (**F**) baseline variables, DMN degrees and FPN degrees.

The most important predictors per predictive model according to SHAP scores are presented in Fig. 3. The top five most important variables per model are shown to preserve visual clarity. Data points to the left of the zero line represent cases in which the feature contributed to a non-impairment prediction, while data points to the right contributed to an impairment prediction. Blue-coloured data points represent low feature values, while red-coloured data points represent high feature values. This means that a distribution of red values on the right means high values predict impairment, while red values on the left mean high feature values predict non-impairment. Conversely, blue values on the right mean low values predict impairment, and blue values on the left mean low values predict non-impairment.

When using baseline variables available at T0 to predict T3 impairment, older age, having a tumour in the right hemisphere, and cognitive impairment at T0 are the most important predictors for impairment at T3 (Fig. 3A). For T0 DMN degrees, we see that low left lateral superior frontal gyrus, right accumbens and right precuneus degrees are important predictors of cognitive impairment (Fig. 3B). When using T0 FPN degrees, low right pars opercularis, right accumbens and left middle frontal gyrus degrees predict impairment (Fig. 3C). When adding baseline variables, older age becomes an important predictor (Fig. 3D and E). Finally, in the model combining all predictors, low right accumbens and left lateral superior frontal gyrus degree, and older age are the most important predictors (Fig. 3F).

The most important predictors when predicting T0 impairment from T0 predictors and predicting T3 impairment from T3 predictors are presented in Supplementary Materials 2 (Supplementary Figs. 1 and 2, respectively).

## Discussion

In this study, we aimed to predict postsurgical cognitive impairment of 63 individual glioma patients using baseline and SC predictors. We found that postoperative cognitive impairment could be significantly predicted by low preoperative SC degrees of ROIs in the DMN and FPN. Importantly, the prediction of postsurgical cognition from baseline variables was improved by adding DMN and FPN degree predictors, and tumour location variables only did not predict impairment.

Previous studies were able to predict postsurgical cognitive performance or impairment in individual glioma patients from ‘postsurgical’ network-related predictors. Friedrich *et al*. predicted the outcomes of seven neuropsychological tests from postsurgical whole-brain SC.^47^ In their study, connectome-based predictive modelling was used^103^ in which the weight of each edge in the whole-brain networks of patients is correlated with neuropsychological scores in order to identify a set of edges that explain impairment. They found that for cognitive tasks, predictive edges were mostly between the two hemispheres, often involving regions of the DMN, specifically the left temporal cortex and bilateral posterior cingulate cortex. Of these regions, we also found poor integrity of the left posterior cingulate and left middle temporal gyrus to be predictive of cognitive impairment in our study. Mrah *et al*. predicted deterioration of a task-switching test [trail-making test (TMT), difference between time taken on TMT A and TMT B] from lesions (based on resection cavities) virtually applied to group-based template connections between Yeo’s functional networks.^104^ They found that resections overlapping with the connection template of Yeo network 12, which is part of the FPN,^26^ significantly predicted postsurgical ‘decline’ in performance. This network overlaps with the supramarginal gyrus, pars opercularis and middle frontal gyrus. Of these regions, we found both the right pars opercularis and the middle frontal gyrus to be involved in postsurgical cognitive impairment.

Our results underline the utility of individualized structural connectomes in predicting cognitive impairment after surgery. Earlier work investigating postoperative cognitive outcomes in glioma patients has demonstrated that traditional clinical and location-based variables do not add predictive value on top of preoperative cognitive measurements.^105,106^ In contrast, our work shows that individual-level SC does improve predictions beyond this level, allowing an additional 5% of patients with and an additional 10% without postsurgical impairment to be correctly identified. Furthermore, our results underscore the importance of connections involving the DMN, FPN and subcortical regions in cognitive functioning 3 months after surgery. In particular, we found the connectivity with the right nucleus accumbens to be an important predictor in almost all models (Fig. 3). The nucleus accumbens is generally found to have a role in integrating the limbic system with the prefrontal cortex (PFC), which contains important FPN and DMN structures.^107,108^ In this context, the accumbens is hypothesized to mediate the drives between these two areas: the emotional or motivational drives from the limbic system versus the control offered by the PFC.^109,110^ The strength of the connections facilitating integration between these two systems might then be critical in task switching, which is an important part of our cognitive screening battery.^111^

Subcortical regions and several regions in the DMN and FPN are so-called ‘hub regions’ in the brain, which are regions that are disproportionately strongly connected in the brain’s network. Specifically, the precuneus, posterior cingulate gyrus and middle temporal gyrus in the DMN, and the middle frontal and superior frontal gyrus in the FPN are consistently identified as hubs in both functional and structural network studies.^112,113^ Network hubs play a central role in the organization of the human brain,^114^ integrating multiple systems and providing top-down control.^115^ Damage to hubs has been linked to pathology and cognitive impairment in many cases, such as the accumulation of β-amyloid in Alzheimer’s disease,^112^ anomalous hub organization in schizophrenia^23^ and the reorganization of hub activity in severe traumatic brain injury.^116^ In computational simulation studies, damage to hub parts of the DMN were found to greatly affect whole-brain FC.^117^

In glioma patients, network hubs in particular have been found to play an important role in cognition as well.^118^ For example, structural hub assortativity, the propensity of high-degree nodes to be connected to other high-degree nodes, is associated with higher cognitive performance in glioma patients 1 year after diagnosis.^119^ One possible parallel with our study is that decreased structural assortativity can be caused by reduced hub connectivity, as a reduction in hub connectivity is statistically likely to be due to weaker hub-hub connections. It has recently been hypothesized that the failure of hubs causes a cascading deterioration of network function at a global level, explaining cognitive dysfunction as a result of global network failure across various pathologies.^120^ Hypothetically, the lesioning of any connection or region can cause activity to be ‘rerouted’ in the network. Due to their central position in the network, this rerouting often involves hub nodes, which will experience an increased load of activity. This activity overload may cause these hubs to fail, at which point the rerouting cascades to other hubs. Indeed, Carbo *et al*. found that increased processing load for hub nodes accurately predicted lower verbal memory performance in glioma patients,^121^ suggesting that an overload for hub nodes is a biomarker for aspects of cognitive impairment. Within this framework, our results suggest that the strength of connections involving (hub) regions in the DMN and FPN are essential in mediating an effective redistribution of activity when part of the brain is lesioned. This could explain why connectivity at T0 is predictive of T3 impairment while tumor location at T0 is not: independent of the location of lesions caused by surgery and adjuvant treatment, the main disruption to global network function after treatment might be caused by a necessary rerouting of activity around these lesions. The strength of connections in the DMN and FPN before surgery might then be a proxy for how well this increased activity can be routed through multiple important hubs in the brain (e.g. the precuneus, posterior cingulate cortex, superior frontal gyrus), which allows the global network to adapt to the instantaneous local damage without overloading more vulnerable hub regions. In this sense, presurgical SC of subcortical regions, the DMN, and the FPN might quantify the network’s general resilience to lesions, possibly related to cognitive reserve.^122^ Specifically, on top of being an important integrator of DMN connectivity, it has recently been hypothesized that the nucleus accumbens, which we found to be an important predictor of postsurgical cognitive impairment, plays an important role in cognitive reserve as a central hub in a network regulating stress coping.^123^ Additional analyses conducted on our data set (Supplementary Materials 2), investigating the predictive power of connections with the Visual Network (VN), confirm that the potential for resilience might be specific to the associative networks (i.e. the DMN and FPN) and their respective subcortical hubs, as connectivity with the sensory VN at T0 was unable to predict cognitive impairment at T3 (Supplementary Table 5). Further evidence for this rerouting hypothesis is provided by repeated intraoperative stimulation studies.^64,124^ By mapping intraoperative cortical stimulation points in LGG patients who were operated multiple times, these studies demonstrated that functions in the cortex dynamically re-organize between surgeries. Clearly, the functional organization of the brain of glioma patients undergoes significant changes after surgery, in which the integrity of connections in the DMN and FPN might play a critical role. With fMRI and/or EEG data available before and after surgery, we might be able to measure reorganization and the hub overloading effect in individual patients, and how it relates to SC of the DMN and FPN and to cognitive impairment after surgery. This additional source of data could be used to further improve our predictive model.

Besides their central role in the network, hubs in the DMN and FPN might be sensitive predictors of cognitive impairment because brain disorders tend to disproportionately affect hubs.^125^ It is hypothesized that the relatively high amount of activity in hubs ‘attracts’ pathological development, which is in line with the migration of glioma along large WN bundles^126^ and recent evidence that gliomagenesis and glioma migration are associated with high activity.^127,128^ Indeed, gliomas have been found to preferentially originate in regions with higher functional hubness.^129^ In this view, gliomas are more likely to occur in or around hubs, which makes it more likely for a glioma and its resection to damage such hubs. Indeed, we observe a high concentration of tumours around the right superior frontal gyrus and right middle temporal gyrus in Fig. 2. Consequently, damage to hubs might be more predictive for cognitive function partly due to the fact that damage is disproportionally afflicted on hubs. Furthermore, due to the high (global) connectivity of hubs, it is simply more likely to damage connections with hubs than connections with non-hubs, which means connectional lesion-symptom mapping is more highly powered for hub nodes.

The importance of older age as a predictor for impairment is remarkable, given that the *Z*-scores we based impairment status on were already corrected for the effect of age on cognitive functioning in a healthy control group. In the group of glioma patients, we may expect that older patients are more strongly affected by the disease than younger patients, due to having a smaller cognitive reserve.^122^ This makes older patients more sensitive to disease burden, but also more sensitive to the effects of surgery and adjuvant therapy, explaining the role of age in predicting impairment at T3.

A number of issues could have limited the outcome of our study. Firstly, a sample size of 63 is fairly small for a machine-learning study. It is reasonable to think that a model trained on a larger patient cohort will have better performance, even though our model already achieved reasonably high performance. Secondly, the validity of tractography in glioma patients is occasionally called into question, mostly due to the high variability in reconstructed tracts as a result of different preprocessing pipelines and tracking methods.^130^ It is possible that the WM and GM signals found by SS3T-CSD inside glioma are artefacts of the method instead of the result of actual WM- and GM-like tissue, making tractography through glioma unfounded. However, the fact that we were able to predict cognitive impairment from tractography performed in and around glioma, both in presurgical (Table 2 and Supplementary Table 2) and in postsurgical (Supplementary Table 3) settings, supports the notion that these signals are informative. Thirdly, since our IDH determination may be inaccurate on elderly patients with grade II tumours according to recent guidelines,^131^ we may have misclassified the tumour grade of two elderly patients in our data set. Finally, we retrospectively included a high number of LGG patients (54%) as compared to the incidence of LGGs in proportion to all glioma grades. This is likely due to our inclusion criteria regarding the availability of diffusion-weighted MRI both preoperatively and postoperatively, which in our centre is more likely to be acquired for LGG patients than for HGG patients.

As a next step, our model based on presurgical connectivity can be improved to achieve higher accuracy, e.g. by including activity information derived from resting-state fMRI to detect hub overloading, and validated in a larger patient sample. Such a model could aid in neurosurgical decision-making by assessing the risk of cognitive impairment at an individual patient level. Furthermore, a larger sample of patients might allow us to consider more granular network features, such as individual connections between ROIs, improving the specificity of our findings. Finally, understanding individual-level cognitive decline after surgery, for example by incorporating information from resting-state fMRI, might allow us to also develop a model to predict cognitive decline based on structural disconnections at individual level. If validated, this could allow the development of a virtual surgery planning, where planned resections can be inscribed into presurgical scans to estimate structural damage and subsequent cognitive deterioration.

## Supplementary Material

fcaf346_Supplementary_Data

## Data Availability

The glioma segmentation neural network was trained on the publicly available BraTS data set.^132,133^ SC matrices, baseline predictors and cognitive test scores are available on Figshare (https://doi.org/10.6084/m9.figshare.c.7578326.v1). Raw patient imaging data are not shared due to privacy concerns.
