# Supplementary material for: Presurgical structural connectivity predicts postsurgical cognitive impairment in glioma patients
Source: Brain Commun. 2025 Oct 24;7(5):fcaf346. doi: 10.1093/braincomms/fcaf346 (PMC12550501; doi:10.1093/braincomms/fcaf346)
Supplement: fcaf346_Supplementary_Data [file fcaf346_supplementary_data.docx]

**S1 Cognitive testing details**

| **Test** | **Description** | **Scoring** |
| --- | --- | --- |
| Verbal memory recognition | Fifteen words are presented in sequence. Subjects are asked to identify these target words among a larger set including new words, both immediately after the initial presentation and at the end of the entire test battery. | Number of items correctly recalled across both conditions |
| Visual memory recognition | Fifteen images are presented in sequence. Subjects are asked to identify these target words among a larger set including new images, both immediately after the initial presentation and at the end of the entire test battery. | Number of items correctly recalled across both conditions |
| Symbol digit coding (processing speed) | Eight symbols are presented along with corresponding numbers. Subjects are asked to assign matching numbers to rows of eight randomly ordered symbols for two minutes. | Number of correct responses minus number of incorrect responses |
| Stroop effect | Colored words are presented to subjects. In congruent trials, subjects are asked to press the space bar if the color of the word aligns with its semantic meaning. In incongruent trials, subjects are asked to press the space bar if the color does not match the semantic meaning. | Average reaction time on correct responses for the incongruent trials |
| Stroop interference |  | (Average reaction time on correct responses for the incongruent trials minus average reaction time on correct responses for the congruent trials) divided by average reaction time on correct responses for the congruent trials |
| Shifting attention (executive function) | Subjects are presented with a blue square and a red circle. Third shapes are presented, and subjects are asked to match the new shape either by color or by shape. | Number of correct responses minus number of incorrect responses |
| Continuous performance | Subjects are presented with a sequence of letters and are asked to only respond to a target letter for five minutes | Average reaction time to the target |
| Motor speed | Subjects press the space bar as often as possible within ten seconds using one hand. The trail is repeated three times for each hand. | Average number of presses across all trails summed for both hands |
| Reaction time | Subjects press the space bar as fast as possible when a word appears on screen for five minutes. | Average reaction time |

Supplementary Table 1: detailed description of cognitive test battery.

**S2 Supplementary analyses**

**Methods**

**Impairment at T0 and connectivity at T3**

We conducted additional analyses on presurgical cognitive and postsurgical imaging data to investigate whether individual connectomes could also be used to predict cognitive impairment at the same time point.

T1 and DWI images were obtained three months after surgery, on the same day as postsurgical cognitive testing. Initially, the same image processing pipeline was used to process postsurgical images. However, we found that SLANT does not perform well on postsurgical scans, possibly due to the presence of large resection cavities. Therefore, we chose to transfer the presurgical segmentations to postsurgical scans for each patient by nonlinearly registering the presurgical T1 to the postsurgical T1 scan and applying the resulting transformation to the segmentations. Manual inspection confirmed that this resulted in a good fit with postsurgical individual patient anatomy.

The same neuropsychological screening battery was administered before surgery, on the same day as presurgical MRI scanning. We defined cognitive impairment at T0 in the same way as cognitive impairment at T3, additionally correcting for practice effects when calculating the Z-scores.

We trained random forest classifiers in the same way as before, using baseline variables available at T0 and structural connectivity degrees at T0 to predict cognitive impairment at T0, and baseline variables available at T3 (including tumor grade and administration of adjuvant therapy) and structural connectivity degrees at T3 to predict cognitive impairment at T3. Again, the top five most important predictors were determined using Shapley additive explanations (SHAP) for tree models.

**Visual network**

We carried out additional analyses to confirm whether the success of connections with the DMN and FPN in predicting postsurgical cognitive impairment as described in the main text are specific for these two networks, rather than resulting from a whole-brain connectivity effect. We investigated whether connectivity at T0 with ROIs in the Visual Network (VN), a network not typically associated with cognitive function, was also predictive of cognitive impairment at T3. Following the same ROI selection procedure as described in the main text, we identified the bilateral calcarine cortex, cuneus, lingual gyrus, occipital pole, occipital fusiform gyrus, inferior occipital gyrus, middle occipital gyrus and superior occipital gyrus as SLANT ROIs belonging to the VN, resulting in a total of 16 predictors in the model. We predicted cognitive impairment at T3 using T0 VN SC only and using a combination of T0 VN SC and baseline variables.

**Results**

The results of predicting T0 impairment from T0 predictors are presented in Table S2. Impairment could be predicted from each set of predictors, with the highest performances achieved by combinations of connectome and baseline variables.

| **Target** | **Predictors** | **AUC (corrected p)** |
| --- | --- | --- |
| T0 2+ impairments |  |  |
|  | Baseline variables | **0.71 (p = 0.006)** |
|  | T0 DMN degrees | **0.70 (p = 0.013)** |
|  | T0 FPN degrees | **0.70 (p = 0.007)** |
|  | Baseline var. + T0 DMN degrees | **0.73 (p = 0.005)** |
|  | Baseline var. + T0 FPN degrees | **0.74 (p = 0.003)** |
|  | Baseline var. + T0 DMN+FPN degrees | **0.75 (p < 0.001)** |

Supplementary Table 2: model performance predicting cognitive impairment at T0 from T0 connectivity and baseline variables.

Prediction accuracy for T3 impairment from T3 predictors is listed in Table S3. The baseline variables (including T0 impairment, tumor grade and administration of adjuvant therapy), DMN degrees and combinations of covariates with DMN degrees and/or FPN degrees achieved significant performance, while FPN degrees alone did not.

| **Target** | **Predictors** | **AUC (corrected p)** |
| --- | --- | --- |
| T3 2+ impairments |  |  |
|  | Baseline variables | **0.70 (p = 0.020)** |
|  | T3 DMN degrees | **0.78 (p < 0.001)** |
|  | T3 FPN degrees | 0.53 (p = 0.425) |
|  | Baseline var. + T3 DMN degrees | **0.77 (p < 0.001)** |
|  | Baseline var. + T3 FPN degrees | **0.66 (p = 0.036)** |
|  | Baseline var. + T3 DMN+FPN degrees | **0.74 (p < 0.001)** |

Supplementary Table 3: model performance predicting cognitive impairment at T3 from T3 connectivity and baseline variables.

| **Target** | **Predictors** | **AUC** |
| --- | --- | --- |
| T3 2+ impairments |  |  |
|  | Baseline + location variables | 0.67 |
|  | T0 DMN degrees + loc. variables | 0.73 |
|  | T0 FPN degrees + loc. variables | 0.73 |
|  | Baseline var. + T0 DMN degrees + loc. Variables | 0.75 |
|  | Baseline var. + T0 FPN degrees + loc. Variables | 0.76 |
|  | Baseline var. + T0 DMN+FPN degrees + loc. variables | 0.74 |

Supplementary Table 4: model performance predicting cognitive impairment at T3 from T0 connectivity, baseline and granular location variables.

Predicting T3 impairment from T0 connectivity in the VN yields no predictive performance above chance level (Table S4).

| **Target** | **Predictors** | **AUC (corrected p)** |
| --- | --- | --- |
| T3 2+ impairments |  |  |
|  | T0 VN degrees | 0.44 (p = 0.78) |
|  | Baseline var. + T0 VN degrees | 0.60 (p = 0.095) |

Supplementary Table 5: model performance predicting cognitive impairment at T3 from T0 connectivity in the visual network and baseline variables.

Using T0 baseline variables to predict T0 impairment, we see that older age, high tumor volume and being female are the most important clinical predictors for cognitive impairment (Figure S1a). When using DMN degrees, a high left medial frontal cortex and right middle temporal gyrus degree, and a low right precuneus degree predict impairment (Figure S1b). In the FPN, low degree of the left pars triangularis and bilateral middle frontal are important predictors (Figure S1c). When baseline variables are included, older age and high tumor volume are still important predictors (Figure S1d&e). When all variables are combined, high left medial frontal cortex degree, low left pars triangularis degree and older age are the most important predictors of impairment (Figure S1f).


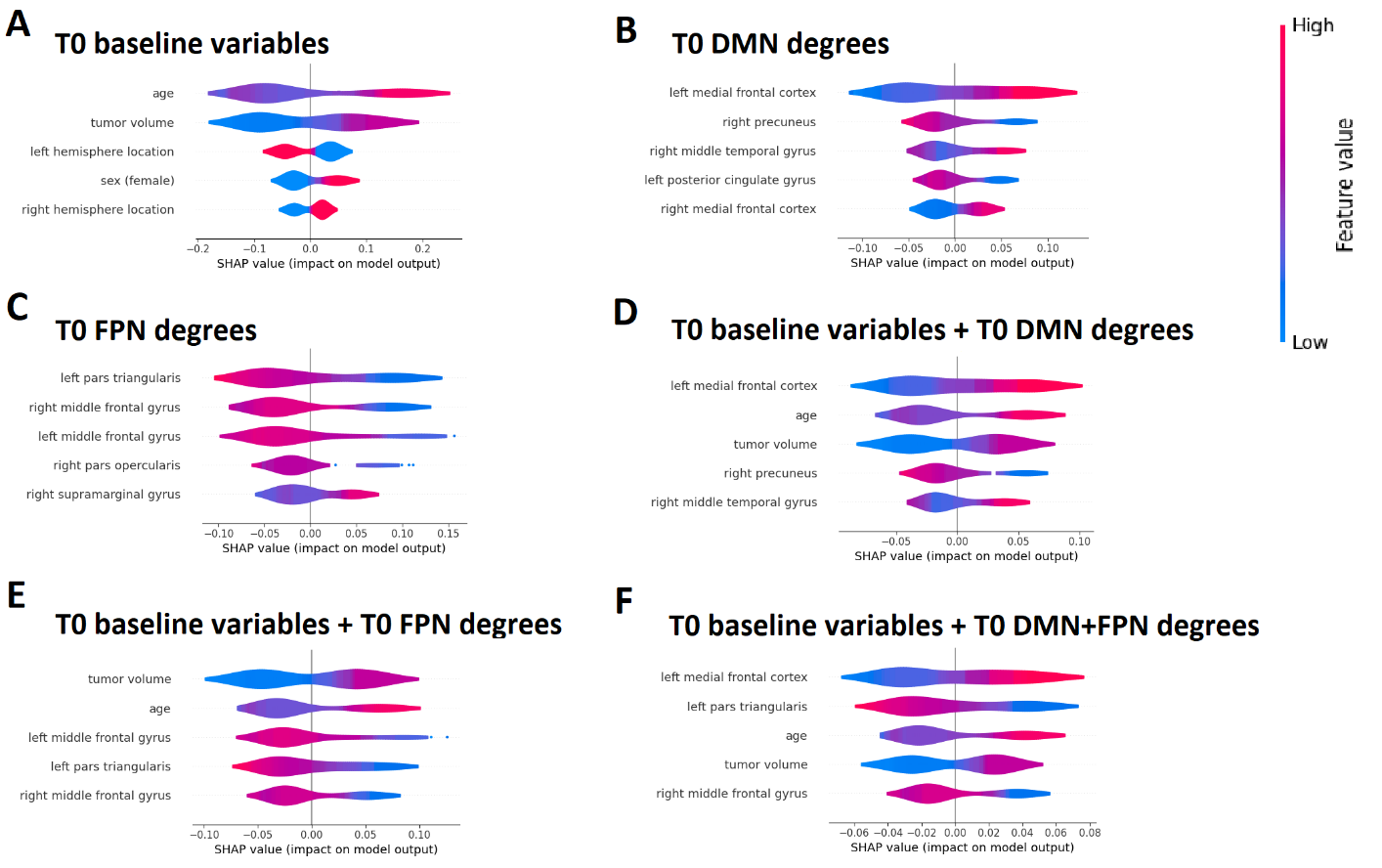


Supplementary Figure 1: SHAP scores for predictor sets that significantly predict T0 impairment from T0 SC degrees.

When using T3 information to predict T3 impairment, we see that older age, right hemisphere location and impairment at T0 are the most predictive baseline variables (Figure S2a). When using T3 DMN degrees, low degree of the right middle temporal gyrus, right posterior cingulate gyrus and right precuneus are important (Figure S2b). When adding baseline variables, older age is also an important predictor for impairment in the DMN model, but right middle temporal gyrus degree and right precuneus degree are more important (Figure S2c). For the FPN and baseline model, we see that older age and right hemisphere location are important, in addition to low right pars opercularis degree (Figure S2d). Finally, in the model combining all predictors, the low right middle temporal gyrus and right posterior cingulate gyrus degrees, and older age are the most important predictors for cognitive impairment (Figure S2e).


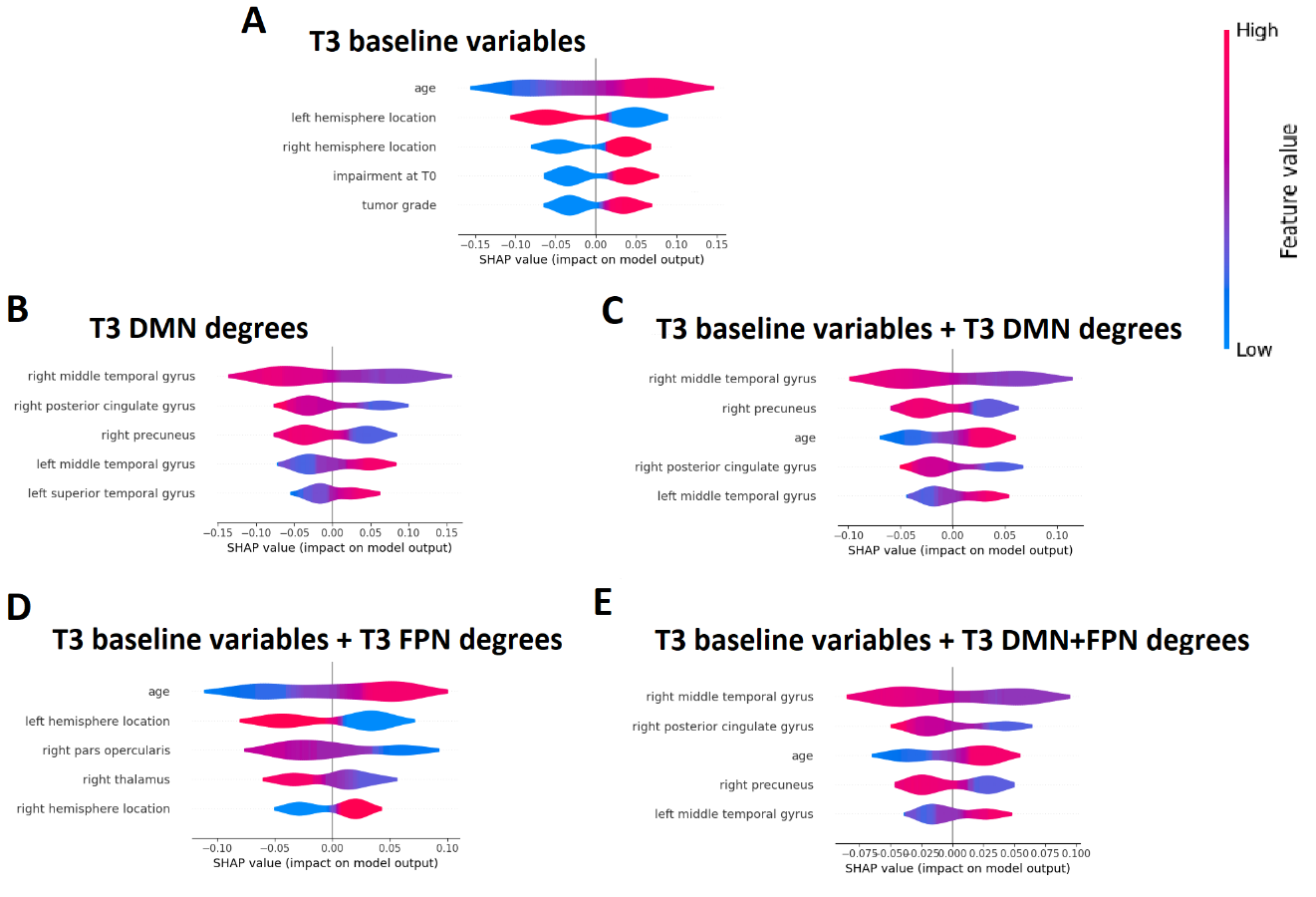


Supplementary Figure 2: SHAP scores for predictor sets that significantly predict T3 impairment from T3 SC degrees.

Surprisingly, high structural connectivity degrees occasionally predicted impairment in our patient group. For example, high left medial frontal cortex and right middle temporal gyrus degree at T0 predicted impairment at T0 (Supplementary Fig. S1). We may hypothesize that high connectivity in these regions indicates a maladaptive reorganization that is detrimental for cognitive functioning. Alternatively, such reorganization might simply indicate a large disease burden that is associated with poor cognitive performance.
